# Flexible vs. rigid dieting in resistance-trained individuals seeking to optimize their physiques: A randomized controlled trial

**DOI:** 10.1186/s12970-021-00452-2

**Published:** 2021-06-29

**Authors:** Laurin Alexandra Conlin, Danielle Trina Aguilar, Gavin Elliot Rogers, Bill I Campbell

**Affiliations:** Performance & Physique Enhancement Laboratory, Exercise Science Program, Department of Educational and Psychological Studies, University of South Florida, 4202 E. Fowler Ave., PED 214, Tampa, FL 33620 USA

**Keywords:** Diet, Diets, Bodybuilding, Physique enhancement, Resistance exercise, Fat loss, Body fat percentage, Muscle mass, Caloric deficit, Exercise

## Abstract

**Abstract:**

**Background:**

The purpose of this study was to compare a flexible vs. rigid diet on weight loss and subsequent weight regain in resistance-trained (RT) participants in a randomized, parallel group design.

**Methods:**

Twenty-three males and females (25.6 ± 6.1 yrs; 170 ± 8.1 cm; 75.4 ± 10.3 kg) completed the 20-week intervention (consisting of a 10-week diet phase and a 10-week post-diet phase). Participants were randomized to a flexible diet (FLEX) comprised of non-specific foods or a rigid diet (RIGID) comprised of specific foods. Participants adhered to an ~20%kcal reduction during the first 10-weeks of the intervention and were instructed to eat ad libitum for the final 10-weeks. Body composition and resting metabolic rate were assessed 5 times: (baseline, 5, 10 [end of diet phase], 16, and 20 weeks).

**Results:**

During the 10-week diet phase, both groups significantly reduced bodyweight (FLEX: baseline = 76.1 ± 8.4kg, post-diet = 73.5 ± 8.8 kg, ▲2.6 kg; RIGID: baseline = 74.9 ± 12.2 kg, post-diet = 71.9 ± 11.7 kg, ▲3.0 kg, *p* < 0.001); fat mass (FLEX: baseline = 14.8 ± 5.7 kg, post-diet = 12.5 ± 5.0 kg, ▲2.3 kg; RIGID: baseline = 18.1 ± 6.2 kg, post-diet = 14.9 ± 6.5 kg, ▲3.2 kg *p* < 0.001) and body fat% (FLEX: baseline = 19.4 ± 8.5%, post-diet = 17.0 ± 7.1%, ▲2.4%; RIGID: baseline = 24.0 ± 6.2%, post-diet = 20.7 ± 7.1%, ▲3.3%; *p* < 0.001). There were no significant differences between the two groups for any variable during the diet phase. During the post-diet phase, a significant diet x time interaction (*p* < 0.001) was observed for FFM with the FLEX group gaining a greater amount of FFM (+1.7 kg) in comparison with the RIGID group (−0.7 kg).

**Conclusions:**

A flexible or rigid diet strategy is equally effective for weight loss during a caloric restriction diet in free-living, RT individuals. While post-diet FFM gains were greater in the FLEX group, there were no significant differences in the amount of time spent in resistance and aerobic exercise modes nor were there any significant differences in protein and total caloric intakes between the two diet groups. In the absence of a clear physiological rationale for increases in FFM, in addition to the lack of a standardized diet during the post-diet phase, we refrain from attributing the increases in FFM in the FLEX group to their diet assignment during the diet phase of the investigation. We recommend future research investigate additional physiological and psychological effects of flexible diets and weight regain in lean individuals.

## Introduction

A majority of weight loss efforts fail to provide long-term weight maintenance [1]. Such outcomes support the contention that some view weight loss as a transient period of time and do not recognize the necessity for permanent lifestyle and dietary habit change. Diet-restricted weight loss creates a host of distinct biological adaptations, including but not limited to increased hunger, decreased satiety, suppressed energy expenditure, and altered levels of circulating hormones known to influence weight loss and maintenance [2, 3]. These adaptations inevitably cause weight regain if permanent lifestyle changes are not created.

To achieve successful weight loss, an individual must develop and maintain dietary patterns that create a caloric deficit [4, 5]. If this vital criterion is not first met, an individual will fail in their weight loss efforts [6, 7]. Optimal approaches to weight loss and weight loss maintenance from a physiological perspective are currently unknown. Regardless of what may or may not be optimal from a physiological perspective, an emerging body of evidence suggests that adherence to a diet, regardless of the type of diet, is an important factor in weight loss success [7, 8].

In addition to understanding the importance of creating a calorie deficit is the recognition that some extent of cognitive restraint must be instilled on the part of the dieter to achieve successful weight loss. Westenhoefer [9] suggested that cognitive restraint can be divided into two categories, identified as ‘rigid control’ and ‘flexible control’. Rigid control typically represents an all-or-nothing approach to an individual’s eating behaviors and attitudes toward dieting, while ultimately (negatively) influencing their weight; while flexible control typically represents a more moderate approach to those behaviors. Individuals adopting rigid control of their diet will eliminate “forbidden” foods from their daily food intake, thereby narrowing the variety of foods ingested [10]. Rigid control is associated with a dichotomous mindset, in that an individual displays an “on or off” mentality and any slight deviation from the plan can cause the individual to switch “off” the diet. This switching off phenomena commonly results in increased levels of disinhibition (overeating), binging, or setbacks [11–13]. Further, rigid restraint allows little to no variation of food choices, has been associated with higher levels of non-planning compulsiveness and less successful weight maintenance [14]. In concert with this, Palascha and colleagues concluded that holding dichotomous beliefs about food and eating may be linked to a rigid dietary restraint, which in turn impedes people's ability to maintain a healthy weight [15].

Alternatively, flexible control is marked by a more moderate approach to dieting behaviors. Previous literature has shown how flexible control positively impacts weight loss, partly from the absence of overeating typically characterized by rigid control. Additionally, it reduces the effect of food cravings and allows the dieter to adjust their daily food consumption without compensatory behaviors that negatively affect long-term weight maintenance [16]. These long-term, flexible dietary behaviors allow for higher levels of self-regulation, less disinhibition and lower binge eating behaviors [14, 17, 18].

For resistance-trained individuals seeking to enhance their physique via reduction of fat mass while maintaining/gaining fat-free mass—best practice guidelines suggest a moderate caloric deficit with relatively high dietary protein intakes [19–22]. With macronutrient (protein, carbohydrate, and fat) targets that provide a high protein intake and sufficient energy to produce moderate energy restriction, the individual can construct their diet to their own preferences, lifestyle, and spontaneous events of life, potentially leading to increased adherence and dietary freedom. A diet that prioritizes individual preferences among bodybuilders and individuals wanting to enhance their physiques is referred to as “If It Fits Your Macros” (IIFYM) [23]. The IIFYM dieting approach, as it is practiced by resistance-trained individuals seeking to optimize their physiques, places an emphasis on strategically targeted macronutrient totals, with an emphasis on higher protein intakes, and as such is also commonly referred to as “macros-based dieting”. This IIFYM/macros-based dieting approach has the purpose of allowing more dietary inclusion and variety versus an inflexible meal plan, and is therefore more consistent with flexible dietary control than rigid dietary control [23]. It should be noted that a macro based, “flexible” diet is not necessarily synonymous with flexible restraint, although it does have promise to be more flexible then a diet with no substitutions.

Recent research has shown the value and increased likelihood of weight loss success and long-term maintenance through adopting a flexible form of restraint while dieting [11–13]. Unfortunately, the current literature on this subject is limited in resistance-trained populations. The primary purpose of this study was to compare the effects of a flexible versus rigid dieting approach in this population. A secondary purpose of the current investigation was to observe weight regain in the post-diet period during an ad-libitum phase. To the best of our knowledge, this is the first dietary intervention to investigate a flexible vs. rigid diet and its effects on body composition and metabolism during a diet (active weight loss) phase and a post-diet (weight regain) phase in a non-obese, resistance-trained population.

## Methods

**Fig. 1:**
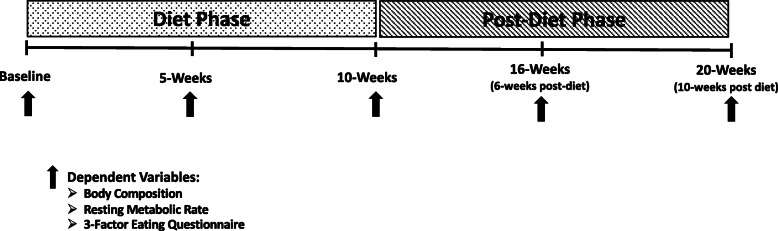
Overview of study phases and testing sessions

A parallel-groups, repeated measures design was utilized for this investigation. A 20-week intervention consisting of a 10-week diet phase and a 10-week post-diet phase was implemented. At baseline, participants were matched according to fat mass and then randomized to follow either a flexible diet or an isocaloric and isonitrogenous rigid diet in conjunction with a resistance and aerobic training program for ten weeks followed by an additional ten weeks of an ad-libitum diet. Participants visited the laboratory on five occasions: baseline, weeks 5 and 10 (midpoint and end of diet phase); and two times during the post-diet/ad-libitum phase (weeks 16 and 20; Fig. 1). Before each laboratory visit the participants were instructed to fast overnight and refrain from physical activity for the previous 24-hours. The primary dependent variables (DVs) of interest were body composition (fat-free mass [FFM], fat mass, and body fat percentage). Secondary DVs included resting metabolic rate (RMR) and measures of eating behavior via the Eating Inventory, also referred to at the Three-Factor Eating Questionnaire (TFEQ).

## Participants

**Fig. 2:**
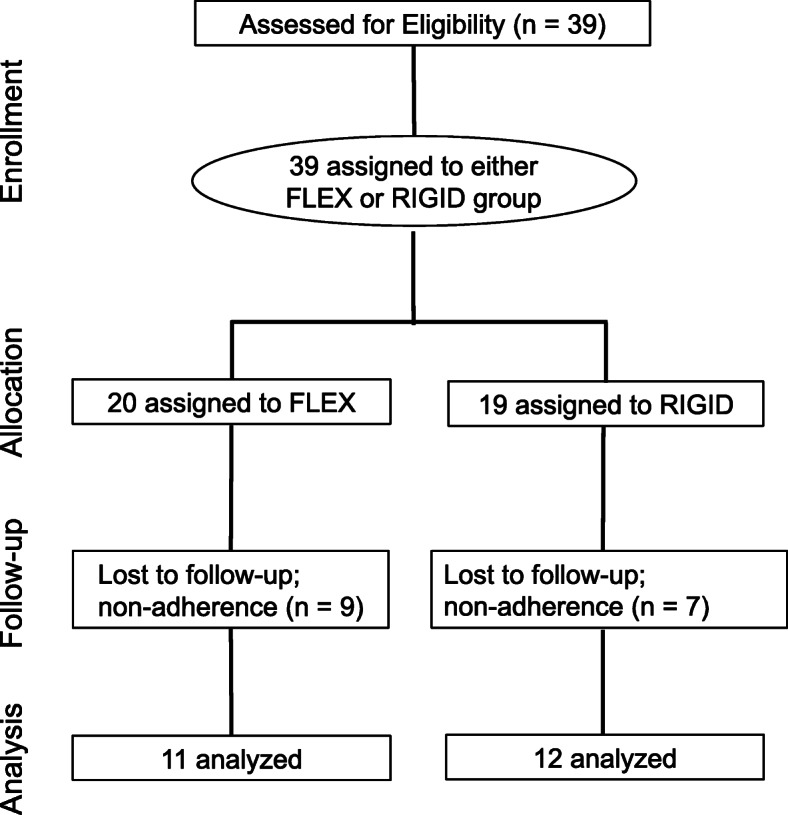
CONSORT participant flow

Thirty-nine healthy, resistance-trained subjects seeking to enhance their physiques were recruited for participation. Twenty-three males (*n* = 10) and females (*n* = 13) aged 18–39 (25.6 ± 6.1 years; 170 ± 8.1 cm; 75.4 ± 10.3 kg) completed all aspects of the intervention. Inclusion criteria required at least one year of prior resistance training experience and a current engagement in a resistance training program for at least two hours per week. All participants provided written informed consent and the Institutional Review Board at the University of South Florida approved the study protocol (Pro00021377). Fig. 2 summarizes the participant study flow.

## Body Composition, Resting Metabolic Rate, and Three-Factor Eating Questionnaire

Upon entering the laboratory, participants urinated and then had their body weight (BW) measured on a physician beam scale (Health-O-Meter, Model 402KL, McCook, IL, USA). Next, body composition was assessed using the Body-Metrix™ BX-2000 A-mode ultrasound (IntelaMetrix, Livermore, CA) with a standard 2.5 MHz probe according to procedures as previously described [24]. All body composition assessments were completed by the same technician whose calculated fat mass test-retest reliability was: ICC 0.99; SEM 0.25 kg (2.6%); minimal difference 0.69 kg.

After body composition assessments were completed, RMR testing procedures were conducted in a manner as previously described [25]. Intra and inter-day test-retest correlation calculated for the device used in the present study were as follows: intra-day RMR Pearson correlation was *r* = 0.96 (*p* < 0.01) and the inter-day RMR Pearson correlation was *r* = 0.90 (*p* < 0.01). Intra-day RMR ICC was 0.981 and the inter-day RMR ICC was 0.946. Following the RMR measurement, participants completed the Three-Factor Eating Questionnaire (TFEQ). The TFEQ is used to assess three dimensions of human eating behavior—cognitive restraint of eating, disinhibition, and hunger [26]. Test-retest reliability was found to be satisfactory for all three dimensions [26]. The TFEQ was administered to each subject at baseline, after the dietary intervention (week 10), and upon the conclusion of the 10-week post-diet period (week 20).

## Dietary and Exercise Intervention

Prior to baseline testing, participants tracked their typical diet for three days (including one weekend day) for determination of maintenance calories. Subjects were instructed to not change their eating habits while keeping the 3-day food record such that the average 3-day caloric intake would serve as an estimaton of maintenance calories. Participants were placed on a diet that prescribed a 25% reduction from their maintenance calories. A moderate caloric deficit was chosen due to the likelihood of participant adherence coupled with its effectivenees to induce fat loss. Previous work from our laboratory (in a similar, resistance-trained population) implemented a 25% caloric deficit and resulted in favorable outcomes relative to body composition changes [25]. In some instances, there was a mismatch between reported maintenance calories and measured RMR (for example, subjects were reporting caloric intakes less than their measured RMR values). In such cases, either a 15% caloric reduction or a 1200-calorie diet was prescribed (whatever was higher in caloric intake was assigned to the participant). This implementation ensured that no subject was prescribed a diet of less than 1200 calories during the 10-week diet phase. These adjustments were evenly distributed among the two intervention groups (four subjects in each group).

Participants were instructed to consume 2g protein/kg body mass and to approximately split their remaining calories evenly between fat and carbohydrate. Subjects were matched according to fat mass and randomly assigned to a flexible (FLEX) or rigid (RIGID) diet plan. The RIGID dieting group was given an individualized set meal plan and were instructed to only eat foods that were included on their specific meal plan and to not make food substitutions. All set meal plans were prepared by a registered dietitian and each subject was provided with a few sample meal plans to choose from at each total daily caloric intake value. Table 1 provides an overview of a sample meal plan for a participant assigned to the RIGID group. The FLEX group was provided with a comprehensive ebook on how to count their macronutrient intakes [27], and were subsequently given macronutrient totals (listed as carbohydrate, protein, and fat grams) to consume each day during the 10-week diet phase. Food choices were not restricted and participants were encouraged to eat a wide variety of food sources. In addition, participants completed a 3-day food record prior to each testing session. Participants were instructed to continue their normal resistance and aerobic training during the study. Further, they were instructed to not change their typical exercise programs during the investigation. Participants were instructed to record the time (start time and end time) that they engaged in resistance training and aerobic training on a training log.

**Table 1:** Sample menu plan for a 60-kg participant.

Meal 1	Carbohydrates (g)	Protein (g)	Fat (g)
¾ cup egg whites	1	20	0
2 large whole eggs	0	12	9
1-ounce avocado	2	0	4
1.25 cups spinach	1.5	1	0
1-piece whole wheat toast	15	3	1
½ of a medium sized banana	12.5	0	0
Total	32	36	14
Meal 2			
4 ounces of chicken breast	0	20	1
¾ cup sweet potato	21	1	0
1 cup broccoli	6	2	0
1 ounce of almonds	4	4	14
Total	31	27	15
Meal 3			
¾ cup fat free Greek yogurt	9	23	0
1 tablespoon peanut butter	4	4	8
1 cup mixed berries	17	1	0
Total	30	28	8
Meal 4			
4 ounces of lean ground beef	0	22	11
1 cup romaine lettuce	2	0	0
½ cup tomato slices	3	0	0
1 ounce of cheese	1	6	9
½ cup brown rice	24	2	1
Total	30	30	21
Total Calories (grams)	492 (123g)	484 (121g)	522 (58g)

Note: 1,500 calorie reduced diet sample menu is based on a 25% reduction from a 2000-baseline calorie maintenance level

## Statistics

Descriptive statistics (M ± SD) for all DVs were calculated. Nutrition data was analyzed via a 2 group (FLEX vs. RIGID) × 3 time (baseline, diet phase, post-diet phase) between-within factorial ANVOA. Body composition and RMR data was analyzed via a 2 group (FLEX vs. RIGID) × 5 time (baseline, mid-point of diet phase, end of diet, mid-point post-diet phase, and end of intervention) between-within factorial ANOVA. Post-hoc tests were conducted with independent samples t-tests by study phase (diet phase or post-diet phase) with appropriate adjustments for alpha levels for multiple comparisons. Baseline differences were analyzed via an independent samples t-test and within-group changes over time were analyzed via a paired samples t-test. For each outcome, an effect size (ES) was calculated as the pretest-posttest change, divided by the pooled pretest SD. We analyzed data per-protocol rather than intention-to-treat since our interest was in the effect of the intervention rather than its prescription. All analyses were completed using SPSS (Version 22, IBM. Armonk, NY) software and the alpha criterion for significance was set at 0.05.

## Results

For body composition and RMR data, a Shapiro-Wilk’s test [28, 29], skewness and kurtosis coefficients, and a visual inspection of their histograms, normal Q-Q plots, and box plots showed that the data were normally distributed (with the exception of diet phase fat mass loss in which skewness *z* score = -2.6; kurtosis *z* score = 2.33) [30]. Dietary intake and behavior data are summarized in Table 2. There were no significant differences between the two diet groups for any dietary intake variable, with the exception of baseline total protein intake. However, when protein intake was adjusted relative to body mass, this difference was negated. The caloric restriction was approximately 20% for both diet groups (20% and 17.7% for the FLEX and RIGID diet groups, respectively). No significant differences between the two diet groups were observed for eating inventory responses.

**Table 2:** Dietary intake and eating behavior data

		Flexible			Rigid	
	Baseline	Diet Phase	Post-Diet Phase	Baseline	Diet Phase	Post-Diet Phase
Macronutrient Data						
Kcal	2289 ± 741	1827 ± 453^*^	2067 ± 433	1904 ± 504	1567 ± 226^*^	1721 ± 318^*^
CHO (g)	235 ± 112	153 ± 44^*^	218 ± 64^*^	181 ± 74	126 ± 41^*^	168 ± 54^*^
PRO (g)	137 ± 39^¶^	153 ± 20	148 ± 37	106 ± 29^¶^	133 ± 40^*^	125 ± 27
Fat (g)	89 ± 36	67 ± 29^*^	67 ± 19	84 ± 33	59 ± 23^*^	61 ± 19
Kcal/kg body mass	30 ± 10	25 ± 7^*^	28 ± 5	25 ± 6	22 ± 4^*^	24 ± 5^*^
CHO (g/kg day)	3.2 ± 1.6	2.1 ± 0.7^*^	2.9 ± 0.9^*^	2.4 ± 0.9	1.7 ± 0.5^*^	2.4 ± 0.8^*^
PRO (g/kg day)	1.8 ± 0.4	2.1 ± 0.2	2.0 ± 0.3	1.4 ± 0.5	1.8 ± 0.5^*^	1.8 ± 0.4
Fat (g/kg day)	1.2 ± 0.5	0.9 ± 0.4^*^	0.9 ± 0.3	1.1 ± 0.4	0.8 ± 0.4	0.9 ± 0.2
CHO/PRO/Fat (%)	41/24/35	34/33/33	42/29/29	38/22/40	32/34/34	39/29/32
TFEQ						
Cognitive Restraint	12.3 ± 5.4	13.6 ± 4.8	12.9 ± 5.7	12.1 ± 4.8	13.2 ± 3.3	11.7 ± 4.2
Disinhibition	6.2 ± 3.0	6.4 ± 3.5	6.1 ± 3.5	6.1 ± 3.0	6.0 ± 3.2	5.4 ± 2.8
Hunger	5.9 ± 2.9	5.5 ± 3.0	6.0 ± 2.7	5.8 ± 3.3	5.7 ± 3.1	5.5 ± 4.2

*CHO* carbohydrate, *PRO* protein, *TFEQ* Three-Factor Eating Questionnaire

^¶^Significant difference between groups at baseline (independent samples *t* test): *p* < 0.05

*Significant difference from previous time point (1-Way ANOVA): *p* < 0.05

**Fig. 3:**
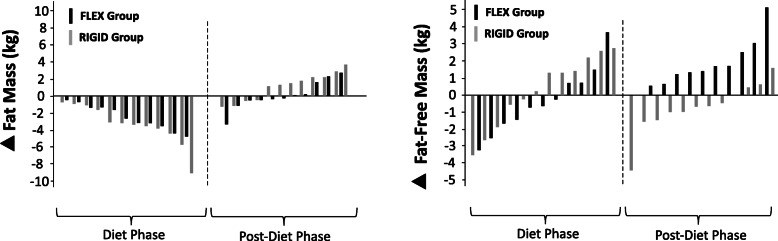
Individual participant changes in fat mass and fat-free mass

There were no significant baseline differences between groups on any measured body composition and RMR variable. Both dietary conditions were effective for inducing weight loss/fat loss and maintaining FFM during the diet phase. However, during the ad-libitum post-diet phase the FLEX group experienced a significant increase in FFM compared to the RIGID group. Body composition and RMR data are summarized in Table 3 and Fig. 3. There were no significant differences between the two groups for total time engaging in resistance training and aerobic training. Specifically, the FLEX diet group engaged in resistance training for approximately 5 ± 1.1 hours/week during the diet phase and 4.5 ± 1.6 hours/week during the post-diet phase while the RIGID group engaged in resistance training for approximately 4.6 ± 1.75 hours/week during the diet phase and 3.6 ± 1.25 hours/week during the post-diet phase. Relative to aerobic activity, the FLEX group completed approximately 42 ± 38 minutes/week during both the diet phase and post-diet phase. The RIGID group engaged in aerobic training for approximately 96 ± 72 and 65 ± 42 minutes/week during the diet phase and post-diet phase, respectively.

**Table 3:** Body Composition and Resting Metabolic Rate (M ± SD) for Diet Phase and Post-Diet Phase

	FLEXIBLE (n=11)					RIGID (n=12)					
Diet Phase	Pre	Mid	Post	▲ (95% CI)	ES	Pre	Mid	Post	▲ (95% CI)	ES	
Body Weight (kg)	76.1 ± 8.4	74.2 ± 8.4	73.5 ± 8.8*	-2.6 (-3.6; -1.5)	0.30	74.9 ± 12.2	73.0 ± 11.5	71.9 ± 11.7*	-3.0 (-5.2; -0.8)	0.25	
FFM (kg)	61.3 ± 11.3	61.1 ± 10.5	61.0 ± 10.4	-0.3 (-1.7; 1.0)	0.03	56.8 ± 10.7	57.1 ± 11.0	57.0 ± 10.8	+0.2 (-1.1; 1.5)	0.02	
Fat Mass (kg)	14.8 ± 5.7	13.1 ± 5.6	12.5 ± 5*	-2.3 (-3.3; -1.2)	0.43	18.1 ± 6.2	15.9 ± 6.5	14.9 ± 6.5*	-3.2 (-4.7; -1.6)	0.50	
Body Fat (%)	19.4 ± 8.5	17.9 ± 7.9	17.0 ± 7.1*	-2.4 (-4.1; -1.1)	0.31	24.0 ± 6.2	21.8 ± 7.3	20.7 ± 7.1*	-3.3 (-4.5; -2.0)	0.50	
RMR (kcals/day)	1816 ± 229	1805 ± 169	1827 ± 257	+11 (-112; 134)	0.05	1576 ± 350	1631 ± 377	1649 ± 375	+73 (-83; 231)	0.2	
Post-Diet Phase	Pre	Mid	Post	▲ (95% CI)	ES	Pre	Mid	Post	▲ (95% CI)	ES	*p*
Body Weight (kg)	73.5 ± 8.8	74.1 ± 9.7	75.2 ± 9.8*	+1.7 (0.2; 3.2)	0.18	71.9 ± 11.7	72.4 ± 10.8	72.3 ± 11.6	+0.4 (-0.9; 1.9)	0.03	0.461
FFM (kg)	61.0 ± 10.4	61.6 ± 10.8	62.7 ± 11.3*	+1.7 (0.8; 2.7)	0.16	57.0 ± 10.8	56.8 ± 10.3	56.3 ± 10.5	-0.7 (-1.6; 0.2)	0.07	0.037
Fat Mass (kg)	12.5 ± 5.0	12.5 ± 4.4	12.5 ± 4.4	0 (-1.1; 1.1)	0	14.9 ± 6.5	15.6 ± 6.4	16.0 ± 6.4*	+1.1 (0.1; 2.1)	0.17	0.562
Body Fat (%)	17.0 ± 7.1	17.2 ± 6.4	17.0 ± 6.6	0 (-1.5; 1.0)	0	20.7 ± 7.1	21.6 ± 7.2	22.1 ± 6.8*	+1.3 (0.03; 2.7)	0.19	0.338
RMR (kcals/day)	1848 ± 261	1850 ± 279	1983 ± 396	+135 (-8; 278)	0.39	1649 ± 375	1749 ± 407	1755 ± 387	+106 (-11; 222)	0.28	0.488

*FFM* Fat-free mass, ▲ Post – Pre, *CI* confidence interval, *ES* Cohen’s d effect size, *RMR*, resting metabolic rate

*p* = 2x5 group x time interaction (post-hoc analysis revealed differences between the diet groups were during the post-diet phase only)

*Significantly different from pre-measures (*p* < 0.05)

## Discussion

This study examined the effectiveness of adopting a flexible versus rigid dietary approach to weight loss during a 10-week diet phase in healthy, resistance-trained males and females. Previous literature has shown that restrictive dieting leads to greater weight gain over time versus adopting a flexible dieting strategy [16, 18, 31, 32]. The primary finding of this study was that a flexible and rigid diet were equally effective for inducing fat loss during the diet phase, but during the post-diet ad-libitum period, the flexible diet group experienced improvements in FFM that were not observed in the rigid diet group.

There are few studies that have investigated non-overweight, resistance-trained individuals undergoing a weight loss regimen for which to make comparisons with our investigation. Garthe et al. [21] compared a slow and fast rate of weight loss in resistance training elite athletes during a similar length of time. Body composition changes were similar in the present investigation as compared to the resistance-training athletes in the Garthe study. Garthe et al. [21] reported greater losses of fat mass and greater gains in FFM with a slower rate of weight loss (0.7%/week) as compared to a faster rate of weight loss (1.4%/week). The rate of weight loss for the FLEX and RIGID groups combined was ~ 0.4%/week. In addition to a slow rate of weight loss, we instructed the subjects to ingest 2g of protein/kg body mass per day during the diet phase. Mettler et al. [22] reported that there was a significantly reduced loss of FFM with the ingestion of high protein intakes (~2.3 g/kg day) compared with a control diet of ~1.0 g/kg day during a short-term diet phase in resistance-trained athletes. The conservative rate of weight loss combined with the relatively high dietary protein consumption likely explains the favorable body composition changes observed for both groups in the present study.

There is an inverse curvilinear relationship between initial body fat content and the proportion of weight loss consisting of lean tissue [33]. It is anticipated, therefore, that a change in weight for a lean individual would elicit a larger relative change in FFM than would be the case for an obese person. Given the relatively lean status of the participants in the current study, it could be expected that composition of body weight lost would be comprised of relatively higher losses in FFM (△FFM/△BW). In contrast, with both groups combined, the loss of body mass was almost entirely accounted for by the loss of fat mass (98% of the body mass lost was from fat mass). This was similar to the combined (slow and fast rate of weight loss) groups from the Garthe et al. [21] investigation in which they observed a body recomposition (all of the weight lost was from fat mass stores and there was a trivial gain of FFM). Our results were also comparable to two other studies in which resistance-trained individuals consumed relatively high dietary protein while in a caloric deficit [22, 34]. Specifically, body fat mass reductions consisted of 67% and 80% of total body mass lost as reported by Pasiakos et al. [34] and Mettler et al., [22], respectively. The preferential reduction of fat mass and the preservation of fat-free mass was likely due to the relatively high protein intake, the slow rate of weight loss, and the inclusion of a resistance training program throughout the diet phase.

The only observed body composition difference between the two dietary groups was during the post-diet/ad-libitum phase in which the FLEX group gained significantly more FFM as compared to the RIGID group. Of those completing the post-diet phase, 10 of 11 (91%) subjects in the FLEX diet group gained FFM, as compared to only 3 of 12 (25%) subjects in the RIGID diet group. Unfortunately, we do not have a strong hypothesis to offer for this observation. There were no significant differences in dietary intakes between the two diet groups during the post-diet/ad-libitum phase. Similarly, there were no significant differences in the amount of time spent resistance and aerobic training during the same time period. While the amount of time engaging in these exercise modes did not significantly differ between the groups, unfortunately we did not collect data relative to the intensity of the loads lifted during the resistance training sessions. It is possible that differences in the relative intensities differed between the groups which may have accounted for some of the difference in fat-free mass that was observed.

Another possible explanation is the connection between dietary restraint and psychological distress [35]. Bartholomew et al. [36] reported that high life stress mitigates a person’s ability to adapt to resistance training. It is possible that the participants assigned to the RIGID group experienced greater levels of stress by having to adhere to a limited dietary intake pattern, thereby compromising their ability to adapt to the resistance training program. In a male bodybuilder case study, it was reported that a rigid approach adopted during the weight loss phase preceded elevations in mood disturbance scores in the post-diet phase [37]. It is important to note that our investigation did not measure psychological distress or mood disturbance, and at this time our recognition of this possible link is theoretical until more research can be conducted into this area.

There were no significant differences in resting metabolic rate between the two groups, suggesting that as long as the caloric restriction is not severe, that relatively high protein is consumed, and resistance training is performed — adverse metabolic adaptations can be minimized regardless of a flexible or rigid dietary approach to caloric restriction [38–40]. Both treatment groups displayed high levels of cognitive restraint during the diet phase. Cognitive restraint measures conscious attempts to monitor and regulate food intake. Prior research in sedentary, overweight females identified cognitive restraint (among other factors) as the most predictive factor of weight change [16]. Disinhibition scores were low for both groups. This dimension of eating behavior assesses uncontrolled eating in response to emotional cues. Hunger scores slightly decreased (non-significantly) in both diet groups. This dimension measures the extent one experiences feelings of hunger in their daily lives. High restraint, low disinhibition, and low hunger scores predict greater weight loss, which is what was observed in our participants, regardless of diet group.

Once again, it should be noted that flexible restraint as a whole is distinct from “macro-based dieting.” Within the resistance trained and physique-minded community, it is not uncommon for macro-based dieting to be a highly rigid dieting practice. While food choice may be autonomous, the rigidity of hitting very specific macronutrient targets can pathologize into a more negative relationship with food and body image. Neither of these pathologies lead to long-term successful weight maintenance and further research is needed to clarify the ideal level of flexibility and rigidity amongst dieting behaviors.

There were several limitations in our study that should be considered. A primary limitation of the study was the subject attrition rate of approximately 40%. Our sample size estimation was 34 participants. While we recruited approximately 5 subjects (13%) above this threshold, our attrition rate was greater than anticipated. Additionally, the number of subjects that dropped out of the study could have affected the randomization of subjects. There was a slightly greater attrition rate in the FLEX as compared to the RIGID group (difference of two subjects). A possible explanation for this was the duriation of the intervention in which participants in the RIGID group felt that the provision of a meal plan was easier to follow because it removed the planning aspect. Also, maintenance calories were calculated from the tracking and recording of three days of food intake (including one weekend day). A longer period of caloric intake tracking would have been better and likely would have provided a more accurate estimation of true maintenance calories. There was also an assumption that all dietary tracking was conducted accurately and honestly, and if not accomplished would serve as an additional limitation. Also, our investigation did not measure psychological distress or mood disturbance, which would have been helpful due to the potential connection of dietary restraint and psychological distress that may have explained the differences in post-diet differences in fat-free mass changes. Further, exercise sessions were not supervised by the research study staff. Previous research has reported that when trainees are supervised (as compared to conducting their workouts on their own) greater improvements in FFM are realized [41]. Another limitation of the present study was the utilization of a two-compartment model for body composition with no corrections for total body water (TBW). Previous research has reported the three-compartment model is more valid than the two-compartment model because it controls for biological variability in TBW [42]. Efforts were made to minimize this limitation by requiring the participants to fast overnight, refrain from physical activity for the previous 24 hours, and urinate prior to body composition assessment. Lastly, our definition of flexible dieting should not be confused with flexible restraint. Restraint may be looked at on a sliding scale; a meal plan is more rigid than a “macro-based” diet but that does not mean that a “macro-based” diet is completely flexible. When taken too far, it can pathologize into what is commonly observed with rigid dieting. Future research should examine the effects of how these two types of diet interplay with habit based, flexible restraint. The ongoing presence of the dichotomy of restraint reveals the need for more research investigating long-term dietary adherence strategies. Despite these limitations, the current study addresses a large gap in knowledge regarding the effectiveness of a flexible dieting strategy in resistance-trained individuals seeking to improve their physique.

## Conclusion

This is the first study of its kind to examine the effects of adopting a rigid versus flexible dieting approach in a healthy, resistance-training population during an acute weight loss intervention. A flexible diet is neither superior or inferior to a more rigid dietary approach relative to fat loss, eating behavior, and RMR. At the conclusion of the 10-week diet phase, the FLEX group experienced significantly greater increases in FFM as compared to the RIGID group. However, there were no differences in the amount of time spent in resistance and aerobic exercise modes nor were there any differences in protein and total caloric intakes between the two diet groups in the post-diet phase. In the absence of a clear physiological rationale for increases in FFM, in addition to the lack of a standardized diet during the post-diet phase, we refrain from attributing the increases in FFM in the FLEX group to their diet assignment during the weight loss phase of the investigation. There is ample opportunity for future research to expand this area of inquiry and address some of the limitations of our investigation. Subsequent work into this area should examine whether a standardized exercise program and post-diet controls leads to similar outcomes as well as the impact that dietary restraint and psychological distress have on adaptations to resistance training.

## Data Availability

The datasets used and/or analysed during the current study are available from the corresponding author on reasonable request.
